# Intersectional Mentorship in Academic Medicine: A Conceptual Review

**DOI:** 10.3390/ijerph21040503

**Published:** 2024-04-19

**Authors:** Jae M. Sevelius, Orlando O. Harris, Lisa Bowleg

**Affiliations:** 1 Department of Psychiatry, Columbia University Irving Medical Center, New York, NY 10032, USA; 2 Community Health Systems, School of Nursing, University of California, San Francisco, CA 94143, USA; orlando.harris@ucsf.edu; 3 Applied Social Psychology, The George Washington University, Washington, DC 20052, USA; lbowleg@email.gwu.edu

**Keywords:** mentoring, intersectionality, health equity, academic medicine

## Abstract

Academic medical institutions seek to recruit and retain a diverse workforce to foster equitable, supportive environments in which early-stage investigators, especially those who are underrepresented in medicine, can thrive. Intersectionality is a critical theoretical framework rooted in Black feminist activism and scholarship that elucidates how power and privilege are differentially structured for groups at different intersectional sociodemographic positions. As a dynamic method of analyzing multiple axes of power and inequality, intersectionality has the potential to offer a critical lens through which to view the mentor–mentee relationship. In this article, we seek to elaborate upon and extend the concept of intersectional mentoring, elucidate its essential components, and explore its application in the context of mentoring early-stage investigators in academic medicine. We propose that intersectional mentorship requires an orientation toward deep cultural humility, lifetime learning about the impact of systemic oppressions on present-day opportunities and experiences of mentees, and changing systems that perpetuate inequities by centering praxis—the application of principles of intersectionality through action to transform power dynamics in academic culture and institutions. Intersectional mentorship can help build a more equitable and representative workforce to advance intersectionally relevant and innovative approaches to achieving health equity.

## 1. Introduction

To end health inequities in the United States and beyond, we need an intersectionally diverse workforce that reflects, includes, and supports people from communities experiencing the most profound inequities. Efforts to achieve health equity must be multilevel while centering the perspectives and experiences of those most impacted by disparities, as interventions developed by and for our communities will be more effective and readily implemented [1]. Acknowledging this, the Association of American Medical Colleges (AAMC) articulates the need to “diversify tomorrow’s doctors” in its strategic plan [2]. To address this need, academic medical institutions are seeking to recruit and retain a more diverse workforce, acknowledging the urgency to create more equitable and supportive environments in which clinical and research faculty, particularly those who are underrepresented in medicine (URM), can thrive.

Academic medicine, according to the AAMC, encompasses “the array of organizations which contribute to the education of physicians and biomedical scientists, and which contribute new knowledge through their research programs”. Patient care is often included in these activities. Academic medical centers employ physicians, nurses, other healthcare providers, research faculty, and trainees, including those working in the social and behavioral sciences. In this article, we aim to review the concept of mentoring in academic medicine and elucidate how an intersectional approach to mentoring can help address inequities in academic medical institutions and beyond [3].

Mentoring is the act of an experienced advisor (i.e., a mentor) training and guiding someone (i.e., a mentee) for the purpose of professional and/or personal growth. Mentorship in academic medicine refers to relationships through which mentees acquire the knowledge, skills, support, and guidance necessary to thrive in their careers. Mentoring programs in academic medicine are increasingly focusing on the needs of URM early-stage investigators (ESIs, i.e., trainees and early-career faculty). For example, in 2006, the University of California, San Francisco established the Faculty Mentoring Program to support recruitment and retention and increase faculty diversity. The program pairs faculty with a “career mentor”, defined as a mentor who provides “overall career guidance and support”, and provides suggestions about how to select such a mentor [4]. While similar mentoring programs are emerging in many academic medical institutions, rarely do they provide adequate training or frameworks for mentors to effectively support mentees who are URM [5]. As part of these efforts, here we use an intersectional lens to review the concept of mentorship in academic medicine. We employ conceptual review methodology to elucidate mentoring approaches and institutional changes that can help to successfully recruit, support, and retain diverse researchers and clinical faculty [3,6]. Unlike systematic reviews, conceptual reviews serve to expand existing concepts by bridging models and theories across disciplines [7]. In this paper, we seek to elaborate upon and extend a model of intersectional mentoring, elucidate its essential components, and explore its application in the context of mentoring ESIs in academic medicine.

The most common, traditional type of mentoring relationship is a one-on-one relationship based on a mutual expectation that the mentor will guide and support the mentee in their professional development within their chosen field [8]. Foundational competencies for mentors include communication and managing the relationship, psychosocial support, career and professional development, professional enculturation and scientific integrity, research development, and investigator development [8]. Other characteristics of good mentors include valuable personal qualities, ability to act as a career guide, commitment to meeting regularly, supporting the mentee’s work/life balance, and role modeling [9]. Overall, a good mentor is able to provide support and guidance in the realms of both scientific and psychosocial development of their mentees to help them develop a successful scientific career [8].

Benefits of mentoring for career advancement of ESIs in academic medicine have been well established [10]. Effective mentoring is associated with higher levels of satisfaction with mentees’ career and work environment [11], greater likelihood of promotion to senior ranks and leadership positions [12], more prolific publication records [13], and greater success in obtaining research grants [14]. Early-career mentorship is also a critical factor in URM clinicians’ choice of an academic career over a clinical one [15]. Despite the positive impact that mentorship can have on URM ESIs, they typically receive less mentoring and support than their majority peers [16,17]. In a review of barriers to and facilitators of mentoring ESIs and URM in health-related research, the most frequently cited barrier was a lack of mentors [10]. Barriers to implementing mentoring programs for URM include a dearth of URM faculty available to serve as mentors, lack of sustainability due to time restrictions or funding, requirement of significant time commitment from mentors, and difficulties addressing institutional challenges faced by URM faculty [16].

While inadequate mentoring is not unique to URM ESIs, it disproportionately impacts those from URM backgrounds. URM mentees often face structural barriers when building a career in academic medicine [18,19]. For example, many URM medical students are the first generation in their family to graduate from college, often rendering the “hidden curriculum” inscrutable. Universities were traditionally established to serve cisgender men, and persisting structural sexism can prevent childbearing ESIs from having their needs met regarding family planning and responsibilities, which are often different from ESIs who are not childbearing [20]. Similarly, sexual and/or gender minority (SGM) faculty often face heterosexism and/or cisgenderism in the academy [21]. An intersectional lens illuminates how these structural inequities manifest in the experiences of individuals. These experiences among ESIs must be understood and addressed in supportive relationships with mentors, as well as within the structures of the institution [22].

## 2. Limitations of the “Mutual Marginality” Approach to Mentoring

Often, many faculty assume that the best mentoring would come from senior faculty who belong to similar groups as their mentees and are matched by racial/ethnic group, gender, SGM identity, or other sociodemographic factors. This approach is sometimes referred to as a “mutual marginality” approach, where mentor and mentee share similar experiences of intersectional marginalization [22]. However, due to existing historical inequities including structural racism, the majority of currently available mentors in academic medicine are white and cisgender men [18,23]. For example, in 2018, Black male and female professors represented just 3% and 4% of full-time faculty, respectively, compared with 40% and 35% of professors who were White men and women [24]. URM ESIs, especially those in predominately white institutions who experience marginalization at the intersections of racism, sexism, cisgenderism, heterosexism, etc., may find that there are few available mentors who share their cultural or social backgrounds, identities, values, and/or academic field [12,25].

This “mutual marginality” approach can be problematic for several additional reasons [22]. First, it is not a given that people with similar demographics or identities will share perspectives and skills relevant to the mentoring relationship. Second, this approach may overburden the few senior URM faculty who are successful despite their experiences of intersectional marginalization, thereby contributing further to the disproportionate burden on URM faculty [5]. While mentorship is an important contribution, it is not valued by most academic institutions on par with other elements of faculty’s research agenda, such as securing research grants or publishing peer-reviewed articles [10]. Finally, relying on a “mutual marginality” approach limits the pool of available mentors. Even if a mentee were able to find a mentor with shared experiences of intersectional marginalization who was a good fit for career mentorship, it is unlikely that one person can meet all of a mentee’s various needs for mentoring. Moreover, these needs change over the course of one’s career [26].

Taken together, the benefits of mentoring for ESIs in academic medicine and the institutional and structural barriers faced by URM faculty reveal an urgent need for a framework for mentoring across intersectional differences, such as White faculty mentoring URM ESIs. While developing foundational mentorship skills is essential, here we posit that there are additional competencies and practices for all mentors to build when working within an intersectional framework.

## 3. Intersectionality as a Framework for Mentoring

Intersectionality is a critical theoretical framework rooted in Black feminist activism and scholarship that elucidates how power and privilege are differentially structured for groups at different intersectional sociodemographic positions [27,28]. Power dynamics in the mentoring relationship are an oft-cited concern, particularly among mentees who experience intersectional oppressions, such as racism and sexism [25,29,30]. Intersectionality is a framework developed to “investigate how intersecting power relations influence social relations across diverse societies as well as individual experiences in everyday life” (p. 221) [31].

As a dynamic method of analyzing multiple axes of power and inequality, intersectionality has the potential to offer a unique lens through which to view mentor–mentee relationships. This is perhaps especially true of mentorship across sociodemographic differences that may particularly impact one’s experiences in academia, such as racial/ethnic group, gender, sexual orientation, gender identity, ability status, and socioeconomic status [22]. An intersectional framework reveals how interlocking power relations (e.g., racism, sexism, classism, *and* heterosexism) construct our perspectives and experiences and seeks to inform practical actions toward the liberation of individuals and groups that are negatively impacted [32]. Intersectional positions can create experiences of both opportunity and oppression, such that a person can experience advantage, disadvantage, or both at the same time [33]. Power imbalances in mentoring relationships can be viewed in terms of seniority and academic hierarchy, where the mentor almost always has power over the mentee by design; however, social capital, privilege, and positionality vary according to the intersectional positions of both mentor and mentee as well as social context [26,34].

**Figure 1:**
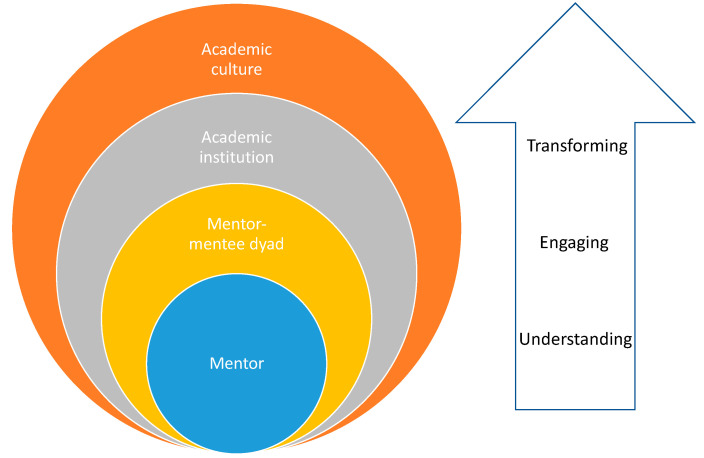
Framework for intersectional mentoring: Addressing intersectional dynamics of power in the mentoring relationship.

In 2020, Brown and Montoya were, to our knowledge, the first to propose a model of intersectional mentorship in academia, which they framed around the issue of sexual harassment [22]. They posit that the mentor’s identity is less important than having an intersectional orientation; that is, mentors must be reflexive about their positionality in relation to others. Further, mentors must be self-aware, oriented toward action to create more inclusive institutions, and work to understand their mentees’ experiences. Here, we propose that a mentor taking an intersectional approach to mentorship in academic medicine aims to *understand*, *engage with*, and *transform* the ways that interlocking systems of power and privilege impact both their own and their mentees’ experiences, the mentoring needs of their mentees, as well as the mentor–mentee dynamic and relationship (see Figure 1) [22,35]. We also identify socio-ecological factors that impact the mentoring relationship and offer several competencies and opportunities for praxis to strengthen the mentor–mentee relationship (see Table 1).

## 4. Understanding Intersectional Dynamics of Social Power in the Mentoring Relationship

Racism, sexism, heterosexism, cisgenderism, ableism, and other systems of structured oppression are critical to interrogate and address, as the intersection of these forces of oppression have had profound, devastating, and far-reaching effects on all aspects of our society [36]. All people belong to multiple social categories (e.g., gender, “race” [37], sexuality), which are both properties of the individual (e.g., identities, behaviors) as well as the social context (e.g., culture, institutions) [38]. To understand dynamics of power in mentoring relationships, intersectional mentorship requires *reflexivity*, i.e., explicit, self-aware analysis of the mentor’s positionality in the mentoring relationship [39]. Mentors must actively reflect upon the multitude of both the mentee’s and mentor’s intersectional positions and how structural forces, such as racism, sexism, *and* cisgenderism, intersect within the context of academic institutions and culture to shape mentoring dynamics, career development, professional relationships, and differential opportunities for mentees.

Because mentoring aims to shape the values, attitudes, and behaviors of mentees [8], the mentor’s self-awareness of their own biases and beliefs is critical to avoiding the reproduction of power dynamics that can limit opportunities for certain mentees. The practice of *reflective praxis*, or the active practice of self-reflection, is most often discussed in the context of qualitative research. However, applying reflexivity to intersectional mentorship can illuminate how the mentor–mentee relationship is co-created and actively shaped by intersectional positions and perspectives of both individuals. Intersectional reflexivity is “thoughtful, conscious self-awareness” (p. 532) [39] that mentors can develop through practices of active examination of their own motivations, assumptions, and interests in mentoring. One potential practice for mentors is taking reflexive notes before and after each mentoring session to reflect on how the mentor is showing up and how their power can impact the mentor–mentee relationship. Mentors can also form peer mentoring groups, where like-minded senior faculty can bring questions and issues about mentoring processes for input from the group. Self-awareness takes practice, time, and commitment, and the willingness to examine intersectional biases when they arise [34,39].

ESIs are more successful when they have mentors who understand and openly acknowledge how one’s social positions shape their experiences of the structural dynamics at play within the institutional environment, as well as within the mentoring relationship itself [17]. Further, intersectional mentorship recognizes that because we all have multiple intersectional positions: some of us may experience privilege in some ways and be unfairly disadvantaged in others [33]. For example, White women may be advantaged due to their color, but not necessarily gender. Thus, intersectional mentors recognize that “racism, sexism, and other systems of structured inequity diminish the strength of the whole society through the waste of human resources” [35]. Intersectional mentors seek to expand their awareness of how privilege and oppression operate to affect themselves, their mentees, their professional relationships, and their institutions.

All mentors must develop the skills to be able to reflect on their own lived experience, acknowledge their biases and privileges, and comfortably explore the lived experiences of their mentees. In this way, mentors let the mentee know that experiences of oppression, such as microaggressions, bias, and discrimination, are legitimate topics for discussion, whether they occur in society or in a professional context [5]. For example, an African American mentee shares his frustrations with his mentor that during patient encounters when—even though he is the attending physician—patients often look to his white trainee when asking questions about their care. Another example is an African American nurse practitioner who, when he steps into a white patient’s room, is addressed as a member of the cleaning staff. Using an intersectional mentoring framework, mentors must be open to engaging in ongoing dialogue to support mentees in successfully navigating these microaggressions. Mentors must be willing to advocate on behalf of their mentees while centering their mentees’ voices, supporting solutions that are defined by the mentees themselves.

## 5. Engaging with Intersectional Dynamics of Power in the Mentoring Relationship

An intersectional approach to mentorship requires deep cultural humility [10]. Cultural humility is a practice in which individuals engage in ongoing self-reflection and self-critique of power, privilege, and inequities within themselves and their relationships [18,40]. Cultural humility requires recognition of the uniqueness of each individual within cultural contexts, thereby acknowledging the importance of tailoring cultural understanding to each individual and relational interaction. For example, a culturally aware mentor will tailor their mentoring style based on the mentee before them, with an understanding that each mentee may experience different inequalities because of their own intersectional positions. Interpersonally, cultural humility entails “being respectful and considerate of the other; being genuinely interested in, open to exploring, and wanting to understand the other’s perspective; not making foreordained assumptions; not acting superior; and not assuming that much is already culturally known about the other” (p. 661) [40,41]. The practice of cultural humility has similarly been encouraged in the cultivation of cultural safety, a concept used in healthcare to describe practices recommended for providers to reduce bias and inequities in the provision of care [42].

An intersectional approach to mentorship requires an orientation toward lifetime learning about how the history of racism and other forms of systemic oppression impact present-day opportunities and experiences of mentees [35,43]. In contrast to a traditional hierarchical approach where the mentor is viewed as the expert and the mentee as the “protégé”, intersectional mentoring acknowledges mutual benefits (e.g., the benefit of the mentee’s labor to the mentor’s research agenda), and structures the relationship to maximize bidirectional learning and minimize toxic power differentials [10]. Without cultivating an orientation toward learning, mentors can inadvertently replicate dominant worldviews and oppressive processes. These oppressive processes create environments where it is discouraged or even dangerous to point out instances of bias and discrimination. In this way, the historical and current intersectional inequities are perpetuated [40]. Acknowledging intersectional differences within the mentoring relationship and practicing cultural humility can prevent dominant values from overtly and covertly pervading the relationship. Avoidance of these issues can create culturally harmful (i.e., unsafe) interactions, ranging from interpersonal microaggressions to the creation of hostile work environments [44].

A critical first step in this study is developing openness to acknowledging and exploring one’s own cultural *inhumility*; for example, cultivating awareness of one’s own biases and personal limitations in understanding intersectional differences. Similarly to the concept of cultural safety [42], cultivating this type of awareness can help limit the impact of mentor biases on their relationships with their mentees. Reflection practices allow the mentor to recognize when these biases arise, refrain from acting upon them, and learn new ways of thinking and being. As mentors provide guidance and feedback, they must also seek guidance and feedback from mentees about their approach and whether their needs are being met in a supportive and productive manner. By learning from their mentees as well as their own experiences about the barriers to opportunity faced by ESIs, mentors can then strive to address these barriers and use their power and privilege to create space and new opportunities.

## 6. Transforming Intersectional Dynamics of Power in the Mentoring Relationship and Beyond

Intersectional mentorship is oriented toward actively changing the systems and structures that perpetuate inequities by centering *praxis*, that is, the practical application of principles of intersectionality through action and advocacy to transform power dynamics in academic culture and institutions. Praxis in intersectional mentoring can be as simple as proactively affirming a mentee’s experience of structural challenges or as complex as working to transform long-standing institutional practices and policies. Senior faculty with a history of NIH funding can also use praxis to advocate for ESIs in the scientific review process [45]. They can also use their power and privilege to advocate, nominate or sponsor their mentees for prestigious fellowships or awards.

The myth of meritocracy maintains the illusion that success in academia is solely a product of individual achievement, rendering invisible the interlocking systems of privilege and oppression that enable or challenge this success. Traditional mentorship frameworks that exclusively focus on helping URM to survive academic medical institutions ignore and leave intersectional forms of oppression in place. This individualistic approach perpetuates oppression and inequality by continuing to place the burden of navigating and challenging systemic injustice on the shoulders of URM faculty and trainees [22]. Intersectional mentoring broadens the focus beyond the individual mentee to challenging systems and structures that drive inequities [6,32]. To do this, mentors must not only acknowledge the role of systems and structures in shaping the experiences of their mentees but also must view these systems and structures as modifiable rather than fixed, and use their positions of power and leadership to address inequities on a structural level [35,46].

Building on Dr. Camara Jones’ “strategies for changing opportunity structures” (p. 346), active interventions critical to intersectional mentorship include breaking down barriers to opportunity as well as building bridges to opportunity [35]. Addressing these barriers and building bridges to opportunity involves the mentor conducting an audit of the mentor–mentee relationship to determine mentorship outcomes (i.e., grants, publications, leadership opportunities). Transformation of the academic culture also requires the mentor to participate on merit and promotion committees to elevate and speak to the importance of mentees conducting community-engaged research, which takes time and might lead to fewer publications than clinical or bench science research.

Breaking down barriers to opportunity includes intervening in important decision-making processes [35]. For example, mentors should not only be aware of, but (where possible) intervene on the pervasive issue of the disproportionate administrative burden often placed on URM faculty. URM clinicians are more likely than non-URM clinicians to engage in DEI leadership, provide outreach to and advocacy for underserved communities, and provide mentorship to URM trainees [47]. Leaders at the departmental and institutional levels must find ways to reduce the pressure put on URM ESIs to represent URM perspectives through extensive service, as well as appropriately measure and compensate this service in the promotion and tenure process (e.g., through paid DEI leadership positions and counting service in tenure decisions). However, even if this service is compensated, it may detract from the ESI’s own research and/or clinical agenda. Senior faculty must also increase the number and range of perspectives that can be included by serving on faculty search committees and actively advocating to ensure an intersectionally diverse candidate pool and consideration of diversity metrics when making hiring decisions.

Building bridges to opportunity can include creating more avenues for ESIs to receive intersectional mentorship. Peer and near-peer mentor development programs can provide additional training and support, which also expands the pool of available and well-trained mentors [48]. For example, the first author, a nonbinary-identified White mentor, created a successful virtual peer group mentoring program for URM ESIs conducting research on transgender health disparities to help address the issue of too few mentors in the field. The group provides a safe space for peers and near-peers to share their experiences in academia, get feedback on their research ideas, and combat the isolation that can come from tokenization, or being the “only one” in their department or research group [25,44]. In another example, the second author advocated for the Black, Indigenous, and other People of Color (BIPOC) peer mentoring program within his school to support BIPOC faculty who reported being ignored and abandoned by the school. The mentoring program is led by a senior faculty who also identifies as a person of color. Members provide mutual guidance and support with salary negotiations, teaching assignments, grant applications, and manuscripts. Since the inception of the group, all members have successfully secured extramural funding (to date, a combined $7.5 million) and have published five collaborative papers.

Sponsorship is another important and direct means of creating opportunities for ESIs [6,49]. Sponsorship is related to but distinct from mentorship, in that a leader or senior faculty member who is serving as a sponsor is actively invested in the career advancement of someone more junior. However, sponsorship is not necessarily about providing mentorship or ongoing one-on-one support to the junior person. Sponsorship entails advocating for and nominating the sponsored junior person for leadership opportunities and awards and the sponsor using their position of power to connect the sponsored person to key networks [49]. Because senior leaders and full professors in academic medicine overwhelmingly represent majority groups (e.g., white, cisgender men), there is an imperative for these leaders to actively prioritize sponsorship of URM faculty throughout every aspect of the institution in order to increase diverse representation [49]. Failure to sponsor URM faculty in positions of leadership or promotion to the full professor rank perpetuates legacies of academic institutional racism, diminishes the success of URM ESIs, and decreases retention of URM faculty.

Finally, building bridges to funding opportunities for research and other academic endeavors is a critical intervention. Funding is a structural barrier for many early-career trainees and faculty, but is especially treacherous for URM faculty. A recent study of grants funded by the National Institutes of Health (NIH) found that Black researchers were significantly less likely to be funded than white researchers, and at least 20% of the disparity was attributable to the fact that URM faculty were more likely to conduct community-based or population health-related research than their white counterparts [50,51]. Universities, and academic medicine departments in particular, must work to intersectionally diversify NIH study sections and review panels, while also broadening funding sources to support the careers of and critical research led by URM faculty and eliminate sources of bias and discrimination [50].

## 7. Conclusions and Future Directions

Intersectional mentorship acknowledges and seeks to understand the impact of social positionality on mentoring dynamics in academic medicine, thereby challenging the myth of meritocracy and extending the focus of mentorship beyond the individual. A shift from the individual level to one that is more structural or institutional will allow us to train our attention on the institutional racism, discrimination, and the inequalities experienced by ESIs. However, to achieve this, we must first acknowledge the importance of the historical context in which these institutions are situated. While this conceptual review of mentoring in academic medicine is focused on the social context of the United States, the intersectional forces of oppression described are not unique. Institutions and academic medicine departments must consider and address their distinct cultures that perpetuate discrimination and exclusion [22,35]. An intersectional approach to mentoring in academic medicine has the potential to directly contribute to dismantling structures of inequity in our institutions to attract, support, and retain a more diverse pool of researchers. In addition to building a more equitable and representative workforce, mentees will develop and advance intersectionally relevant and innovative approaches to major health challenges, thereby strengthening our collective efforts to achieve health equity.

## Figures and Tables

**Table 1:** Critical competencies and praxis for understanding, engaging with, and transforming intersectional dynamics of power in the mentoring relationship.

Socio-Ecological Mentoring Levels	Relationship to Intersectional Dynamics of Power in the Mentoring Relationship	Competencies	Praxis
Mentor	Understanding	Understanding how one’s intersectional social positions shape their experiences of the institutional environment as well as the mentoring relationship (e.g., experiences of microaggressions, implicit bias) Ability to mentor with cultural humility	Actively reflecting on own lived experience in relation to social power and position Acknowledging one’s own biases and privileges Through self-awareness, actively seek opportunities for continues learning to identify cultural blind spots that might impact the mentoring relationship
Mentor–mentee dyad	Understanding, engaging with	Creating space and opportunities for the mentor–mentee relationship to evolve into new ways of relating Actively engaging with and understanding the evolving needs of the mentee throughout mentor/mentee relationship	Inviting open, ongoing dialogue with mentees about their experiences of the mentor/mentee relationship. Solicit feedback from the mentee on ways of improving the mentor/mentee relationship.
Academic institution	Engaging with	Ability to structure the mentoring relationship to minimize harmful power dynamics Ability to comfortable explore lived experience of mentees Ability to support mentees in navigating experiences of bias and discrimination	Inviting open, ongoing dialogue with mentees about their experiences of oppression Acknowledge mutual benefits of the mentoring relationship (e.g., mentee has access to mentor’s expertise and networks, mentor benefits from the labor of the trainee, both may learn new skills and content from each other)
Academic culture	Transforming	Ability to work collaboratively with mentees toward transforming harmful institutional practices	Advocating on behalf of mentees while centering mentees voices Contributing to decision-making processes, e.g., serving on faculty recruitment committees to ensure consideration of diversity metrics Creating opportunities for mentees and other URM investigators, e.g., helping them to build a network of mentors, providing sponsorship, creating new training programs with the needs of diverse investigators in mind, identifying funding opportunities to support URM investigators (e.g., NIH diversity supplements)

## Data Availability

Data sharing is not applicable to this article.
